# Clinical Characteristics, Complications, and Outcomes of Hypervirulent Klebsiella pneumoniae Liver Abscess: A Systematic Review of 53 Cases

**DOI:** 10.7759/cureus.96056

**Published:** 2025-11-04

**Authors:** Kohei Oka, Ryuichi Ohta, Chiaki Sano

**Affiliations:** 1 Community Care, Unnan City Hospital, Unnan, JPN; 2 Community Medicine Management, Shimane University Faculty of Medicine, Izumo, JPN

**Keywords:** ageism, diabetes mellitus, general medicine, hypervirulent klebsiella pneumoniae, klebsiella pneumoniae, liver abscess, pyogenic, treatment outcome, virulence factors

## Abstract

Hypervirulent *Klebsiella pneumoniae* (hvKp) is an emerging cause of pyogenic liver abscess (PLA), characterized by virulence genes, such as rmpA and rmpA2, as well as capsular types K1/K2 that enhance mucoviscosity and invasiveness. Although regionally predominant in East Asia, cases are increasingly reported worldwide. We reviewed case reports and case series published between 2000 and 2025 describing hvKp-related PLA with individual-level data. Clinical, microbiological, and outcome variables were summarized descriptively due to heterogeneity across reports. Fifty-three patients from 34 studies were included, mainly from Japan, South Korea, and China. The mean age was 66.2 years, and 81% were male. Diabetes mellitus was the most frequent comorbidity (57%). Reported virulence factors included rmpA (43%), magA (25%), and K1 (43%) or K2 (19%) serotypes. Metastatic infection occurred in 36% of cases, most commonly endophthalmitis (36%), followed by pulmonary (19%) and central nervous system involvement (15%). No carbapenemase-producing strains were identified; most isolates remained broadly susceptible. Third-generation cephalosporins (53%) and carbapenems (26%) were the main therapies, often combined with percutaneous drainage (45%). Overall mortality was 6%, and 23% experienced permanent vision loss. hvKp liver abscess generally carries a low mortality but a high risk of disabling metastatic complications. Early ophthalmologic evaluation and vigilant surveillance for dissemination are essential, and recognition of virulence markers distinguishes hvKp PLA from multidrug-resistant *Klebsiella pneumoniae* infections.

## Introduction and background

*Klebsiella pneumoniae* is a Gram-negative bacillus traditionally recognized as an opportunistic pathogen responsible for nosocomial infections, such as pneumonia and urinary tract infections [1]. However, over the past few decades, a distinct pathotype, hypervirulent *Klebsiella pneumoniae* (hvKp), has emerged, particularly in East Asia, and has increasingly been reported worldwide [2]. Unlike classical *K. pneumoniae *(cKp), hvKp possesses a unique combination of virulence factors, including regulators of mucoid phenotype (e.g., rmpA, rmpA2), capsular polysaccharide synthesis genes (e.g., magA), and siderophore systems that confer enhanced iron acquisition capacity [3]. These features contribute to the hypermucoviscous phenotype, greater invasiveness, and the ability to cause severe infections even in previously healthy individuals.

Among hvKp-associated infections, pyogenic liver abscess (PLA) is the most prominent clinical manifestation [4]. In contrast to PLA caused by cKp or other organisms, hvKp-related PLA frequently leads to metastatic infections, such as endogenous endophthalmitis, meningitis, pulmonary abscesses, and brain abscesses [5]. Such metastatic complications are associated with devastating consequences, including blindness, neurological impairment, and death [5]. Diabetes mellitus has been identified as a significant risk factor, with hyperglycemia predisposing patients to both the onset and dissemination of hvKp infection [6].

Although several systematic reviews and meta-analyses have examined hvKp liver abscess, most were limited by smaller datasets, heterogeneous definitions of hvKp, or aggregated (study-level) analyses without detailed individual patient information. In contrast, the present review provides an updated synthesis encompassing studies published up to 2025, emphasizing case-level data extraction to integrate demographic, microbiological, and clinical outcome details. This approach allows for a more granular understanding of hvKp-related liver abscess, including molecular determinants, regional variation, and patient-level prognostic factors, thereby complementing and extending prior work in this field.

A systematic review focusing on hvKp-associated PLA is therefore warranted. By consolidating evidence from individual patient-level reports, this study aims to clarify the demographic and clinical characteristics of affected patients, the frequency and nature of metastatic infections, treatment modalities including drainage strategies, and patient outcomes such as mortality, relapse, and long-term sequelae.

## Review

Methods

Study Design

This study was conducted as a systematic review in accordance with the Preferred Reporting Items for Systematic Reviews and Meta-Analyses (PRISMA) 2020 guidelines [7]. Our objective was to identify and synthesize evidence from case reports and case series describing clinical features, treatment, and outcomes of PLA caused by hvKp. The study protocol was developed a priori and registered in the International Prospective Register of Systematic Reviews (PROSPERO) (ID number: CRD420251158241).

Data Sources and Search Strategy

A comprehensive literature search was performed using PubMed, Embase, and Web of Science from January 2000 to June 2025. Additional searches of the Cochrane Library and Scopus were conducted when necessary. The search strategy combined controlled vocabulary (MeSH/Emtree) and free-text terms, including "hypervirulent Klebsiella pneumoniae" OR "hvKP" OR "hypermucoviscous" AND "liver abscess" OR "hepatic abscess" OR "pyogenic liver abscess". No filters were applied for study design at the search stage. Reference lists of the included articles and relevant reviews were manually screened to identify additional eligible studies. Only studies published in English were included, although Japanese case reports were considered for supplemental analysis if sufficient clinical details were provided.

Eligibility Criteria

We included case reports and case series describing *K. pneumoniae* liver abscesses that met the clinical or microbiological criteria for hypervirulence. Because molecular testing was inconsistently performed across published literature, inclusion was not restricted solely to genetically confirmed cases. When molecular data were unavailable, hvKp was defined based on clinical presentation (community-acquired, invasive, or metastatic infection in a non-immunocompromised host) and microbiological phenotype (positive string test indicating hypermucoviscosity), consistent with prior definitions in the literature [4-6].

When available, molecular confirmation (e.g., detection of rmpA, rmpA2, or magA or identification of K1/K2 capsular serotypes) was recorded and prioritized for analysis. Cases that lacked both phenotypic (string test) and molecular confirmation were excluded unless the reporting authors clearly described them as hvKp, with justification based on clinical invasiveness. This dual approach allowed the inclusion of representative cases while maintaining diagnostic transparency and reproducibility.

Study Selection

Two reviewers independently screened titles and abstracts for eligibility. Full texts of potentially relevant articles were then assessed in detail. Any disagreements were resolved through discussion or adjudication by a third reviewer. The selection process was documented using a PRISMA flow diagram, ensuring transparency and reproducibility.

Data Extraction

Data were independently extracted by two reviewers using a standardized data collection form. Extracted variables included the following: author, year of publication, country, patient demographics (age, sex), comorbidities (particularly diabetes mellitus), presenting symptoms, laboratory and imaging findings, microbiological and molecular characteristics of hvKp (e.g., rmpA, magA, capsular serotype, sequence type), treatment details (antibiotics, drainage procedures, adjunctive therapies), and outcomes (mortality, recurrence, metastatic infections, and sequelae such as vision loss or neurological impairment).

Data Synthesis

Given the case-based nature and heterogeneity of the included studies, we performed a narrative synthesis of the extracted data. Clinical features, microbiological characteristics, treatments, and outcomes were summarized descriptively, using absolute numbers and proportions where applicable. Although our initial protocol included plans for quantitative pooling, the heterogeneity and incomplete reporting of individual-level data precluded formal meta-analysis. Therefore, findings are presented qualitatively, with emphasis on patterns of metastatic infection, antimicrobial susceptibility, and clinical outcomes. This approach ensures methodological transparency while accurately reflecting the available evidence base.

Results

Study Selection

**Figure 1:**
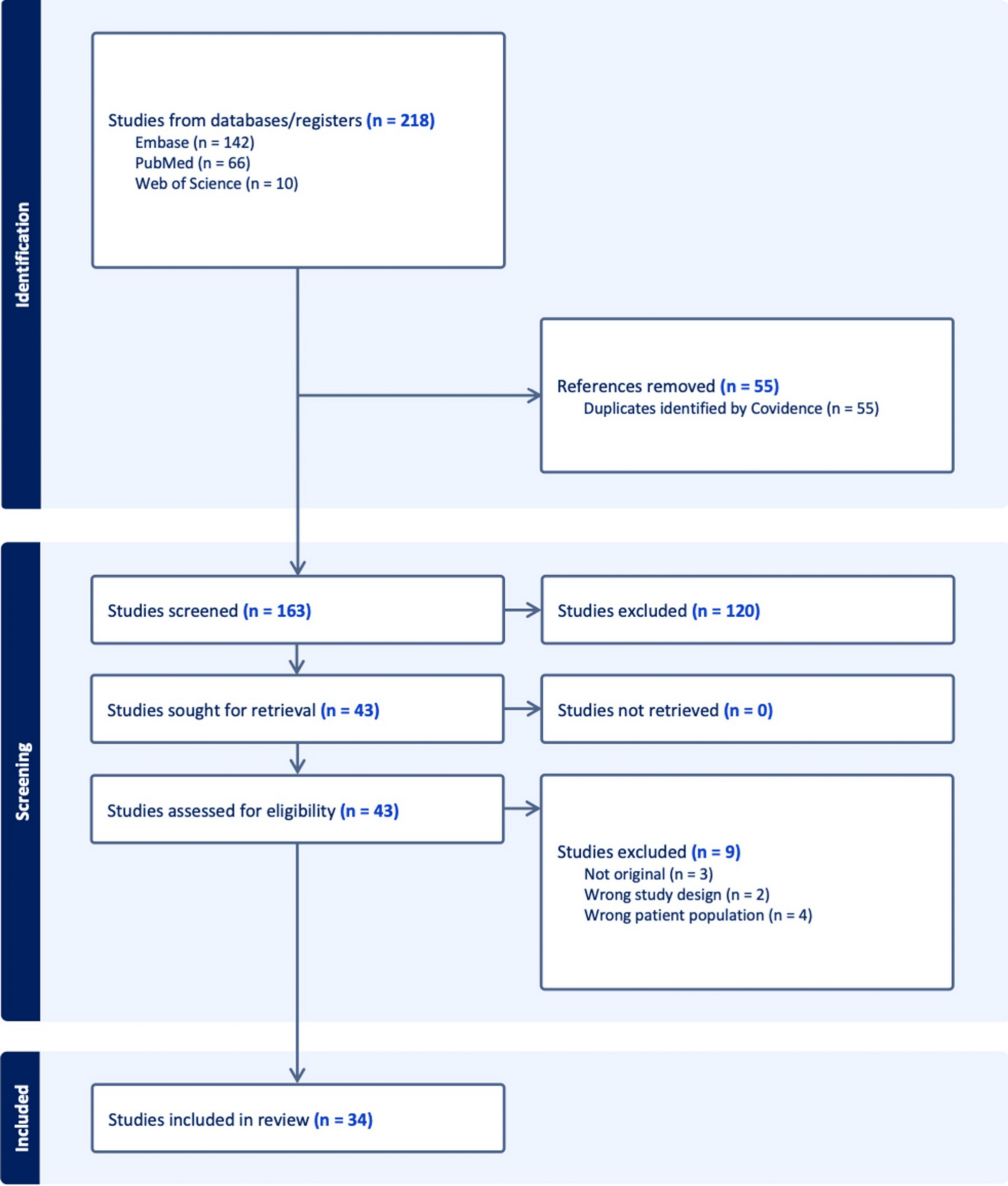
Selection flow

The initial search across three databases (Embase, PubMed, and Web of Science) yielded 218 records (Embase (n=142); PubMed (n=66); Web of Science (n=10)). After removing 55 duplicates, 163 unique records remained for screening. Following title and abstract screening, 43 articles were sought for full-text review. Of these, nine studies were excluded: three were not original research, two had inappropriate study designs, and four did not involve the relevant patient population. Ultimately, 34 studies met the eligibility criteria and were included in the systematic review. No studies were excluded due to retrieval issues, and all potentially relevant full texts were accessible for evaluation (Figure 1).

Overview of the Included Studies

A total of 53 cases of hvKp liver abscess were identified from 34 studies published between 2012 and 2025. Reports originated predominantly from East Asia, particularly Japan (n=14), South Korea (n=11), and China (n=6), reflecting the recognized regional burden of hvKp. Additional cases were reported in Europe (France, Switzerland, Denmark) and North America, highlighting the global spread of this pathogen. Most publications were single-patient case reports, supplemented by a smaller number of case series, underscoring the scarcity of large observational cohorts (Table 1).

**Table 1:** Overview of the included studies "Metastatic infection" indicates the secondary spread of *Klebsiella pneumoniae* beyond the liver, including ocular, central nervous system, pulmonary, or other distant sites. "Outcome" represents patient status at the last follow-up, categorized as "Alive" or "Death". "Recurrence" denotes the documented reappearance of liver abscess or hvKp infection after the completion of initial therapy and clinical resolution. Symbols used in the table were as follows: "+" = present/yes; "-" = absent/no; and "=" = not reported or unclear. Cases are listed by author and publication year, with multiple entries possible from the same study if individual patient data were available. Age is reported at presentation. Sex is recorded as documented in the original report. hvKp: hypervirulent *Klebsiella pneumoniae*

Author/year	Country	Age	Sex	Metastatic infection	Outcome	Recurrence
Enani and El-Khizzi, 2012 [8]	Saudi Arabia	46	Male	+	Alive	-
Enani and El-Khizzi, 2012 [8]	Saudi Arabia	56	Male	-	Alive	-
Merlet et al., 2012 [9]	France	52	Male	=	Alive	-
Gundestrup et al., 2014 [10]	Denmark	85	Male	-	Alive	+
Namikawa et al., 2016 [11]	Japan	81	Male	+	Alive	-
Babouee Flury et al., 2017 [12]	Switzerland	45	Male	+	Alive	-
Babouee Flury et al., 2017 [12]	Switzerland	74	Male	+	Alive	-
Pichler et al., 2017 [13]	Germany	61	Male	+	Alive	-
Scapaticci et al., 2017 [14]	Italy	83	Male	-	Alive	-
Van Keer et al., 2017 [15]	Belgium	84	Male	+	Alive	-
Baekby et al., 2018 [16]	Denmark	78	Male	+	Alive	-
Harada et al., 2019 [17]	Japan	50	Male	-	Alive	-
Harada et al., 2019 [17]	Japan	70	Male	+	Alive	-
Harada et al., 2019 [17]	Japan	60	Male	-	Alive	-
Harada et al., 2019 [17]	Japan	70	Male	-	Alive	-
Hoashi et al., 2019 [18]	Japan	68	Female	+	Alive	+
Hoashi et al., 2019 [18]	Japan	45	Female	-	Alive	-
Imai et al., 2019 [19]	Japan	65	Female	-	Alive	-
Pillsbury et al., 2019 [20]	USA	70	Male	+	Alive	-
Baron et al., 2020 [21]	France	42	Male	+	Alive	-
Baron et al., 2020 [21]	France	60	Male	-	Alive	-
Baron et al., 2020 [21]	France	76	Male	-	Alive	-
Hosoda et al., 2020 [22]	Japan	71	Male	+	Death	-
Kim et al., 2020 [23]	Republic of Korea	50	Male	-	Alive	-
Kim et al., 2020 [23]	Republic of Korea	31	Female	-	Alive	-
Kim et al., 2020 [23]	Republic of Korea	74	Male	-	Alive	-
Kim et al., 2020 [23]	Republic of Korea	70	Male	-	Alive	-
Kim et al., 2020 [23]	Republic of Korea	75	Male	+	Alive	-
Kim et al., 2020 [23]	Republic of Korea	58	Male	-	Alive	-
Kim et al., 2020 [23]	Republic of Korea	91	Female	-	Alive	-
Kim et al., 2020 [23]	Republic of Korea	71	Female	-	Alive	-
Kim et al., 2020 [23]	Republic of Korea	82	Male	+	Alive	-
Kim et al., 2020 [23]	Republic of Korea	47	Male	-	Alive	-
Kim et al., 2020 [23]	Republic of Korea	75	Male	-	Alive	-
Kimura et al., 2020 [24]	Japan	69	Male	+	Alive	-
Sandoval et al., 2020 [25]	Philippines	51	Male	+	Alive	-
Zhao et al., 2021 [4]	China	80	Male	+	Alive	-
Cai et al., 2022 [26]	China	67	Male	+	Alive	+
de Lajartre et al., 2022 [27]	France	67	Male	+	Alive	-
de Lajartre et al., 2022 [27]	France	27	Male	-	Alive	-
Li and Zhang, 2022 [28]	China	78	Female	+	Alive	-
Nayak et al., 2022 [29]	India	48	Male	+	Alive	-
Niimi et al., 2022 [30]	Japan	68	Male	+	Alive	-
De Francesco et al., 2023 [31]	Italy	56	Male	+	Alive	-
Infante-Fernández et al., 2023 [32]	Colombia	77	Male	+	Alive	+
Luo et al., 2023 [33]	China	71	Male	+	Death	+
Zhang et al., 2023 [34]	China	90	Female	-	Alive	-
Ando et al., 2024 [35]	Japan	65	Female	+	Alive	-
Jauvain et al., 2024 [36]	France	80	Male	+	Alive	+
Kalangi et al., 2024 [37]	USA	66	Female	+	Alive	-
Kawase et al., 2024 [38]	Japan	76	Male	+	Alive	-
Chen et al., 2024 [39]	China	72	Male	+	Alive	-
Oka et al., 2025 [40]	Japan	82	Male	+	Death	+

Patient Demographics

Across the 53 identified cases, the mean patient age was 66.2 years (range: 27-91 years), indicating that hvKp liver abscess primarily affects older adults, although younger individuals may also be susceptible. The majority of patients were male (81%), with only 10 female cases reported, consistent with previous observations of a male predominance in PLA.

Diabetes mellitus emerged as the most common comorbidity, frequently associated with poor glycemic control and regarded as a major risk factor for hvKp dissemination. Other reported conditions included cardiovascular disease, malignancy, hypertension, dyslipidemia, and chronic liver disease. In some patients, immunosuppressive therapies (e.g., chemotherapy, corticosteroids, methotrexate) may have contributed to increased susceptibility. Notably, several cases occurred in individuals without underlying comorbidities, underscoring the unique virulence of hvKp compared with cKp. This finding emphasizes that hvKp can cause severe disease even in previously healthy hosts (Table 2).

**Table 2:** Demographic characteristics of patients with hvKp liver abscess (n=53) Values are presented as numbers (%) unless otherwise indicated. Age is presented as mean (range). Some patients had more than one comorbidity; therefore, percentages may not sum to 100%. hvKp: hypervirulent *Klebsiella pneumoniae*

Characteristic	Value
Age, mean (range), years	66.2 (27-91)
Sex, n (%), male	43 (81.1%)
Sex, n (%), female	10 (18.9%)
Diabetes mellitus	30 (56.6%)
Cardiovascular disease	14 (26.4%)
Malignancy	7 (13.2%)
Dyslipidemia	4 (7.5%)
Chronic liver disease	6 (11.3%)
Immunosuppression/therapy	1 (1.9%)

Bacterial Features

Diagnostic confirmation methods varied across the 53 included cases. All patients underwent microbiological culture, most commonly from blood or liver abscess aspirates. The string test was reported in a large proportion of cases, with many showing the characteristic hypermucoviscous phenotype. However, reliance on the string test alone was limited, and several isolates with invasive disease tested negative.

Molecular investigations were performed in a subset of cases. Among those tested, rmpA was detected in at least 10 patients, while magA positivity was observed in a smaller number, typically in K1 isolates. Aerobactin and other siderophore systems were frequently reported, supporting their role in hvKp virulence. Capsular serotyping revealed that K1 and K2 serotypes predominated, while rare lineages such as K64-ST1764 were also described.

In terms of antimicrobial susceptibility, most isolates were pan-susceptible, consistent with classical hvKp phenotypes. Only a minority exhibited resistance, including extended-spectrum β-lactamase (ESBL) production in isolated cases, while no carbapenemase-producing strains were identified. These findings underscore both the heterogeneity in diagnostic approaches and the importance of integrating molecular and phenotypic methods for accurate hvKp characterization (Table 3).

**Table 3:** Microbiological and genetic characteristics of hvKp isolates in liver abscess cases (n=53) Values are presented as number (%). Molecular features were reported only for a subset of cases, and percentages are calculated using the total cohort (n=53) as denominator. hvKp: hypervirulent *Klebsiella pneumoniae*; ESBL: extended-spectrum β-lactamase

Characteristic	Value
Culture performed	53 (100%)
String test positive	21 (39.6%)
String test negative	2 (3.8%)
rmpA positive	23 (43.4%)
magA positive	13 (24.5%)
Aerobactin positive	15 (28.3%)
Capsular serotype K1	23 (43.4%)
Capsular serotype K2	10 (18.9%)
Capsular serotype K64	0 (0%)
Pan-susceptible isolates	36 (67.9%)
ESBL-producing isolates	4 (7.5%)
Carbapenemase-producing isolates	3 (5.7%)

Patient Clinical Features

Among the 53 reported cases, fever was the most common presenting symptom, documented in 33 patients (62%). Abdominal pain was reported in nine cases (17%), often associated with hepatomegaly or localized tenderness. General malaise or fatigue was described in another nine patients (17%), reflecting the nonspecific systemic nature of the disease at onset. In many instances, these symptoms persisted for several days before diagnostic imaging confirmed the presence of liver abscesses.

Metastatic infections were a striking hallmark of hvKp infection. Ocular involvement, including endogenous endophthalmitis, occurred in 19 patients (36%), frequently leading to irreversible visual impairment or blindness. Central nervous system manifestations, such as brain abscess or meningitis, were identified in eight cases (15%), while pulmonary complications, including pneumonia or septic pulmonary emboli, were noted in 10 patients (19%). Less frequently, infective endocarditis (n=3; 6%), psoas abscess (n=2; 4%), and spinal epidural involvement (n=2; 4%) were reported (Table 4).

**Table 4:** Clinical features and metastatic infections in hvKp liver abscess cases (n=53) Values are presented as a number (%). Percentages are calculated using the total cohort (n=53) as the denominator. hvKp: hypervirulent Klebsiella pneumoniae; CNS: central nervous system

Clinical feature	Value
Fever	33 (62.3%)
Abdominal pain	9 (17%)
Malaise/fatigue	9 (17%)
Metastatic lesions	-
Ocular involvement (endophthalmitis)	19 (35.8%)
CNS involvement (brain abscess/meningitis)	8 (15.1%)
Pulmonary complications (pneumonia/septic emboli)	10 (18.9%)
Infective endocarditis	3 (5.7%)
Psoas abscess	2 (3.8%)
Spinal epidural involvement	2 (3.8%)

Treatment

Most patients received broad-spectrum intravenous antibiotics, with third-generation cephalosporins used in 28 cases (53%), often as first-line therapy. Carbapenems were administered in 14 patients (26%), usually for severe or resistant infections, while β-lactam/β-lactamase inhibitor (BL-BLI) combinations such as piperacillin-tazobactam or ampicillin-sulbactam were employed in 13 cases (25%). Additional regimens included metronidazole (n=15; 28%), quinolones (n=13; 25%), and aminoglycosides (n=4; 8%), often as adjuncts.

Drainage was a mainstay of therapy: percutaneous drainage was performed in 24 patients (45%) and surgical drainage in five cases (9%), while 10 patients (19%) were managed without drainage, usually due to small or inaccessible abscesses. Adjunctive interventions included vitrectomy, enucleation, or intravitreal antibiotics for ocular involvement and neurosurgical procedures in patients with central nervous system abscesses (Table 5).

**Table 5:** Antimicrobial therapy and drainage approaches in hvKp liver abscess cases (n=53) Values are presented as a number (%). Adjunctive interventions were reported qualitatively and not consistently quantified across studies. hvKp: hypervirulent *Klebsiella pneumoniae*; BL-BLI: β-lactam/β-lactamase inhibitor

Treatment modality	Value
Cephalosporins (third generation)	28 (52.8%)
Carbapenems	14 (26.4%)
BL-BLI combinations	13 (24.5%)
Metronidazole	15 (28.3%)
Quinolones	13 (24.5%)
Aminoglycosides	4 (7.5%)
Percutaneous drainage	24 (45.3%)
Surgical drainage	5 (9.4%)
No drainage	10 (18.9%)
Adjunctive interventions (e.g., vitrectomy, enucleation, neurosurgery)	Several cases (qualitative data only)

Outcome and Prognosis

The overall outcome was favorable, with the majority of patients recovering after appropriate antimicrobial therapy and drainage. Only three deaths (6%) were reported among the 53 cases, typically occurring early during hospitalization due to septic shock, intracranial complications, or disseminated infection. The remaining patients survived, often with good short-term recovery following treatment. Early diagnosis and timely abscess drainage were associated with improved outcomes, whereas patients who developed disseminated infection were more likely to experience adverse courses.

Although the overall mortality was low (three deaths, 6%), hvKp liver abscesses frequently resulted in significant long-term morbidity. The most common complication was vision loss, observed in 12 patients (23%), often due to endogenous endophthalmitis. In most of these cases, visual impairment was irreversible, and several patients required invasive interventions such as vitrectomy or enucleation. Neurological sequelae were less frequent but clinically important, occurring in two patients (4%) with central nervous system or spinal involvement, leading to persistent disability.

In contrast, patients without metastatic spread typically achieved complete recovery following antimicrobial therapy and drainage, with no major functional impairments reported. These findings indicate that while hvKp liver abscess has a relatively favorable survival rate, it is associated with a high burden of disabling complications, particularly blindness. This underscores the importance of early recognition, aggressive management of ocular symptoms, and close monitoring for metastatic infection (Table 6).

**Table 6:** Clinical outcomes and prognosis of hvKp liver abscess cases (n=53) Values are presented as a number (%). Percentages are calculated using the total cohort (n=53). Prognostic outcomes are described among survivors (n=50). hvKp: hypervirulent Klebsiella pneumoniae; CNS: central nervous system

Outcome/prognosis	Value
Survived	50 (94.3%)
Death	3 (5.7%)
Vision loss (endophthalmitis-related)	12 (22.6%)
Neurological sequelae (CNS/spinal)	2 (3.8%)
Complete recovery (no major sequelae)	36 (67.9%)

Discussion

Summary of the Study

This systematic review demonstrates that PLA caused by hvKp is associated with severe complications, particularly metastatic infections such as endogenous endophthalmitis and central nervous system involvement. However, mortality was relatively low, and long-term morbidity, especially irreversible vision loss, was common. Importantly, our findings suggest that with timely and comprehensive management, including appropriate antimicrobial therapy and drainage, favorable outcomes can be achieved across diverse patient populations regardless of age or comorbidities. This underscores the need for general physicians to provide intensive, patient-centered care that respects individual preferences and avoids ageist assumptions while prioritizing the preservation of quality of life. Our results are consistent with previous studies describing hvKp as a highly invasive but antibiotic-susceptible pathogen, whose severity stems from virulence determinants rather than resistance mechanisms.

Comparison With Other Studies

While our review demonstrates generally favorable outcomes among reported hvKp liver abscess cases, these observations are descriptive and not based on comparative data [3,41]. Therefore, we interpret this trend cautiously, suggesting that timely antimicrobial therapy and drainage may contribute to improved outcomes across patient groups, though further controlled studies are needed to confirm these associations [42]. This aligns with recent case-based evidence showing improved survival with early drainage and intensive supportive care. Nevertheless, our findings reinforce that older adults and patients with multimorbidity remain more vulnerable, experiencing higher rates of morbidity and enduring complications [43,44]. These observations highlight the duality of hvKp PLA as both a potentially curable infection and one with devastating long-term consequences when metastatic spread occurs. Similar demographic patterns have been noted in recent regional analyses, which continue to show a predominance in older men with diabetes mellitus and an increasing number of cases reported outside East Asia [45,46]. The global spread is thought to reflect travel-associated transmission and clonal dissemination of K1 and K2 serotypes, emphasizing the importance of molecular surveillance and international awareness.

Strengths of the Study

This study provides novel insight by synthesizing case-level evidence across diverse populations, thereby highlighting that PLA caused by hvKp can be curable even in complex clinical contexts. Our findings demonstrate that, with early recognition and appropriate interventions such as targeted antimicrobial therapy and drainage, favorable outcomes are achievable not only in younger or otherwise healthy individuals but also in older adults and patients with multiple comorbidities [47]. This observation challenges the assumption that frailty or advanced age inevitably portends a poor prognosis, emphasizing instead the critical role of equitable, aggressive management [48,49]. In particular, it underscores the ethical imperative to avoid therapeutic nihilism and age-based discrimination, which may unjustly limit access to intensive care [50-52]. By situating hvKp PLA within a broader discourse on fairness in medical treatment, this review advocates for a patient-centered strategy that integrates prompt diagnosis, comprehensive management, and long-term functional preservation for all.

Limitations of the Study

Several limitations should be acknowledged to contextualize the strength of the evidence. First, the review is based predominantly on case reports and small case series, which are inherently susceptible to publication and selection bias, as atypical or severe presentations are more likely to be reported. This limits the ability to infer population-level incidence or risk. Second, the geographic concentration of included studies in East Asia reflects the regional predominance of hvKp but also restricts generalizability to regions such as Europe, Africa, and the Americas, where diagnostic awareness and strain distribution may differ. Third, there was substantial heterogeneity in diagnostic criteria across reports: some studies relied solely on the string test. In contrast, others incorporated molecular confirmation using rmpA, magA, or K1/K2 capsular typing, hindering comparability and potentially confounding conclusions about virulence and outcomes. Fourth, incomplete reporting of comorbidities, microbiological findings, and long-term outcomes introduces information bias that cannot be corrected retrospectively.

Furthermore, due to data heterogeneity, a quantitative meta-analysis could not be conducted, limiting inference about prognostic factors. To enhance methodological transparency, we retrospectively evaluated included reports using the Joanna Briggs Institute checklist for case reports, confirming variability in data completeness across studies. These limitations collectively underscore that the evidence is moderate at best and that future multicenter, standardized studies with molecular confirmation are needed to refine the epidemiologic and prognostic understanding of hvKp liver abscess.

## Conclusions

hvKp liver abscess is a distinctive clinical entity characterized by high metastatic potential but generally favorable survival when managed promptly with targeted antimicrobial therapy and adequate drainage. However, substantial morbidity, particularly vision loss, remains a key concern. Given the heterogeneity of diagnostic criteria and reporting, future studies should prioritize standardized molecular confirmation of hvKp, early ophthalmologic screening protocols, and the establishment of multicenter registries to evaluate prognostic factors and long-term functional outcomes systematically. While our findings illustrate trends across age groups and comorbidity profiles, these observations are descriptive rather than comparative and should not be interpreted as evidence of equivalent outcomes. The principal clinical implication is the need for timely recognition and integrated management guided by consistent diagnostic and follow-up frameworks rather than patient demographics.
